# Effects of Metformin as Add-On Therapy against Glioblastoma: An Old Medicine for Novel Oncology Therapeutics

**DOI:** 10.3390/cancers14061412

**Published:** 2022-03-10

**Authors:** Laura Guarnaccia, Stefania E. Navone, Matteo M. Masseroli, Melissa Balsamo, Manuela Caroli, Silvia Valtorta, Rosa M. Moresco, Rolando Campanella, Luigi Schisano, Giorgio Fiore, Valentina Galiano, Emanuele Garzia, Giuseppe C. Appiani, Marco Locatelli, Laura Riboni, Giovanni Marfia

**Affiliations:** 1 Laboratory of Experimental Neurosurgery and Cell Therapy, Neurosurgery Unit, Foundation IRCCS Ca’ Granda Ospedale Maggiore Policlinico, 20122 Milan, Italy; laura.guarnaccia@policlinico.mi.it (L.G.); stefania.navone@policlinico.mi.it (S.E.N.); matteo.masseroli@unimi.it (M.M.M.); melissa.balsamo@policlinico.mi.it (M.B.); manuela.caroli@policlinico.mi.it (M.C.); campanella.rolando@gmail.com (R.C.); luigi.schisano@policlinico.mi.it (L.S.); giorgio.fiore@unimi.it (G.F.); marco.locatelli@policlinico.mi.it (M.L.); 2 Department of Clinical Sciences and Community Health, University of Milan, 20122 Milan, Italy; 3 Department of Medicine and Surgery and Tecnomed Foundation, University of Milan Bicocca, 20900 Milano, Italy; silvia.valtorta@ibfm.cnr.it (S.V.); rosa.moresco@unimib.it (R.M.M.); 4 Department of Nuclear Medicine, San Raffaele Scientific Institute, IRCCS, 20132 Milano, Italy; 5 Institute of Bioimaging and Molecular Physiology, National Research Council (IBFM-CNR), 20054 Segrate, Italy; 6 Reproductive Medicine Unit, Department of Mother and Child, San Paolo Hospital Medical School, ASST Santi Paolo e Carlo, 20142 Milan, Italy; valentina.galiano@asst-santipaolocarlo.it (V.G.); emanuele.garzia@aeronautica.difesa.it (E.G.); 7 Aerospace Medicine Institute “A. Mosso”, Italian Air Force, 20138 Milan, Italy; 8 Italian Air Force Logistic Command, 00185 Rome, Italy; giuseppe.ciniglioappiani@aeronautica.difesa.it; 9 Department of Medical-Surgical Physiopathology and Transplantation, University of Milan, 20122 Milan, Italy; 10 “Aldo Ravelli” Research Center, 20142 Milan, Italy; 11 Department of Medical Biotechnology and Translational Medicine, LITA-Segrate, University of Milan, 20054 Milan, Italy; laura.riboni@unimi.it; 12 Clinical Pathology Unit, Aerospace Medicine Institute “A. Mosso”, Italian Air Force, 20138 Milan, Italy

**Keywords:** glioblastoma, metformin, brain tumors, angiogenesis

## Abstract

**Simple Summary:**

Glioblastoma is the most common and malignant primary brain tumor, with a median survival of around 14 months. The aggressiveness of glioblastoma is due to intense cell proliferation, angiogenesis, invasiveness, genetic instability, resistance to therapies and high frequency of relapses. These features render glioblastoma almost incurable, considered an extreme therapeutic challenge. In the last few decades, it has been observed a reduced cancer incidence in diabetic patients treated with metformin, an oral hypoglycemic drug. The reported ability of metformin to arrest cancer cell growth in in vitro and in vivo experimental tumor models, have suggested the possibility to reconsider metformin as an anti-cancer add-on therapy, but further investigations about molecular mechanisms and optimal therapeutic regimens are needed. Here, we tested the efficacy of metformin against primary glioblastoma endothelial cells, responsible for tumor angiogenesis, invasiveness and resistance to therapy, reporting promising results and advancing a novel target of metformin, the “sphingolipid rheostat”.

**Abstract:**

Background: Glioblastoma is the most aggressive primary brain malignancy in adults, with a poor prognosis of about 14 months. Recent evidence ascribed to metformin (MET), an antihyperglycemic drug, the potential to reduce cancer incidence and progression, but the molecular mechanisms underlying these effects need to be better investigated. Methods: Here, we tested the efficacy of MET on *n* = 10 primary glioblastoma endothelial cells (GECs), by viability and proliferation tests, as MTT and Live/Dead assays, apoptosis tests, as annexin V assay and caspase 3/7 activity, functional tests as tube-like structure formation and migration assay and by mRNA and protein expression performed by quantitative real-time PCR analysis (qRT-PCR) and Western Blot, respectively. Results: Data resulting revealed a time- and μ-dependent ability of MET to decrease cell viability and proliferation, increasing pro-apoptotic mechanisms mediated by caspases 3/7. Also, MET impacted GEC functionality with a significant decrease of angiogenesis and invasiveness potential. Mechanistically, MET was able to interfere with sphingolipid metabolism, weakening the oncopromoter signaling promoted by sphingosine-1-phosphate (S1P) and shifting the balance toward the production of the pro-apoptotic ceramide. Conclusions: These observations ascribed to MET the potential to serve as add-on therapy against glioblastoma, suggesting a repurposing of an old, totally safe and tolerable drug for novel oncology therapeutics.

## 1. Introduction

Glioblastoma, *IDH*-wild type (here named glioblastoma) is the most malignant and frequent glial tumor in adults, classified as grade IV by World Health Organization (WHO) guidelines [1].

Glioblastoma alone accounts for 12–15% of all primary central nervous system (CNS) tumors and 45.8% of malignant ones [2]; nevertheless, its annual incidence is about 3/100,000 people, but increases with age, reaching 15/100,000 people/year between age 75 and 84 [3]. Survival is inversely correlated with age: 5% of all patients who receive a diagnosis of glioblastoma are alive after 5 years, and this value decreases to 2% considering the population aged over 65 years [4]. Median survival ranged from 14.6 to 26.3 months among patients enrolled in clinical studies [5,6].

The therapeutic management of patients with newly diagnosed glioblastoma is currently performed according to the Stupp protocol [7], and consists of the gross total surgical removal of the tumor mass, followed by adjuvant radiotherapy with concomitant, and subsequently maintained chemotherapy with Temozolomide (TMZ).

A main feature of glioblastoma is its intense angiogenesis, the formation of novel disorganized blood vessels, which sustain tumor growth and infiltration into surrounding tissues, finally compromising patient’s neurological skills. Angiogenesis is mediated by tumor endothelial cells, called glioblastoma endothelial cells (GECs), which are strictly involved in glioblastoma resistance to therapies, through the modulation of a sensitive balance between anti- and pro-angiogenic factors [8,9].

In the last few years, several studies have reported a worsened prognosis and a decreased survival in patients affected by both glioblastoma and hyperglycemia [10]. Whether they are linked to a pre-existing diabetes mellitus or corticosteroid therapy is unknown.

In this context, high glucose and insulin levels have been widely described as adverse prognostic factors in many tumors, as breast, colon and prostate cancer [11]. From this evidence, it emerged the potential to use drugs with the capability to lower circulating glucose levels, as adjuvant treatment in cancer patients [12]. Among different drugs, metformin (*N*,*N*-dimethylbiguanide, MET), an oral antihyperglycemic drug of the biguanide family, is widely used as the first line of therapy for all patients with type-2 diabetes mellitus (T2DM) [13]. Of interest, MET exerts significant preventive and beneficial anti-cancer effects in diabetic patients, suggesting its possible use as add-on therapy in many cancer subtypes, including glioblastoma [14].

Several studies performed in different tumor models, including breast, pancreatic, colon, endometrial, ovarian and lung cancer, demonstrated the ability of MET to inhibit tumor cell proliferation, both as monotherapy or in combination with chemo- and radiotherapy [15,16,17]. This effect has also been described in glioblastoma models, where MET proved to inhibit differentiation, invasiveness, autophagy and apoptosis, also in combination with standard therapy as TMZ [18,19,20].

Recent evidence ascribed to MET the ability to also interfere with sphingolipids metabolism and signaling. In particular, in patients suffering from Poly-Cystic Ovary Syndrome (PCOS) and treated with MET, a change in lipidomic signature and metabolism, including glycerolipids, glycerophospholipids, and sphingolipids was observed [21]. The targeting of the sphingolipid system as a therapeutic direction for glioblastoma has been promisingly investigated, since representative oncopromoter as sphingosine-1-phosphate (S1P) and its kinases (SPHK1 and SPHK2) have been clearly recognized as key actors of tumor progression and aggressiveness [22,23,24,25,26]. However, the potential link between metformin treatment and sphingolipids has never been addressed in brain tumors, which makes it interesting to demonstrate if metformin can influence sphingolipid metabolism toward a pro-apoptotic signaling in tumor cells.

With this aim, we here tested the effect of MET on primary GECs, isolated and purified from patient-derived tumor biopsies. Our results prove for the first time a time- and μ-dependent ability of MET to inhibit cell growth, proliferation, invasiveness, and angiogenesis, adding to previous studies the translational relevance of patient-specific ex-vivo models. Furthermore, PCR and protein analyses indicate the sphingolipid rheostat as a molecular mechanism involved in MET action in GECs, further sustaining the potential use of MET in glioblastoma therapy.

## 2. Materials and Methods

### 2.1. Tumor Samples Processing and GEC Isolation

Tissue samples (*n* = 10) from patients that underwent surgical resection for newly diagnosed glioblastoma at the Neurosurgery Unit of Fondazione IRCCS Ca’ Granda Ospedale Maggiore Policlinico (Milan, Italy) were included in the study. The clinical and molecular features of patients and respective glioblastoma tissues are provided in Table 1. The Institutional Review Board approved the protocol (IRB#1670/2015) and all patients provided informed consent. All research was performed in accordance with relevant guidelines and regulations. Glioblastoma biopsies were processed as previously described and GECs were isolated and routinely characterized for endothelial markers [8,9]. GBM cells from tissue biopsies were isolated by the same tumor processing protocol and cultured in Dulbecco’s Modified Eagle Medium/Nutrient Mixture F-12 + 10% Fetal bovine serum (FBS) (both purchased by Thermo Fisher Scientific, Waltham, MA, USA) in a humified 37 °C, 5% CO_2_ incubator.

### 2.2. Pharmacological Treatment

In order to evaluate the effect of MET on GECs and GBM cells, alone or in combination with TMZ, treatments were administered at different doses, with different timing, as follows: (i) MET at 5 mM, 10 mM, 20 mM, and 50 mM, (ii) TMZ 200 μM, (iii) TMZ + MET at 5 mM, 10 mM, 20 mM, and 50 mM. We previously identified the optimal concentration of TMZ on GECs in previous studies [8,9].

### 2.3. MTT Assay

3-(4,5-Dimethylthiazol-2-yl)−2,5-Diphenyltetrazolium Bromide (MTT) assay was used to assess cell viability as a function of redox potential. GECs and GBM cells were seeded (5 × 10^3^/well) in 96-well plates and cultured for 24 h. Then, culture media were replaced with fresh media containing the specific treatments. Tests were performed in triplicate following 72 h of treatment. At the end of treatment, culture media were replaced with 100 μL of fresh media + 10 μL of MTT (5 mg/mL in D-PBS) and, after 4 h of incubation, the media were removed and cells were lysed with 100 μL of 2-propanol/formic acid (95:5, by vol.) for 10 min. Absorbance was read at 570 nm with a microplate reader.

### 2.4. Estimation of Proliferation Rate

GECs (15 × 10^4^) were seeded into 25 cm^2^ collagen-coated flask and cultured in basal conditions or with specific treatments. At 24 h, 48 h, and 72 h, GECs were detached by TrypLE Select, stained with Trypan Blue (ThermoFisher) and counted in a Fuchs Rosenthal counting chamber, to evaluate growth rate. An estimation of viable and non-viable cells was performed at the end of treatment (72 h).

### 2.5. Live and Dead Assay

Live and dead cells were determined by the LIVE/DEAD^®^ Viability/Cytotoxicity Assay Kit, which provides a two-color fluorescence (Calcein AM and EthD-1) cell viability assay by measuring intracellular esterase activity and plasma membrane integrity. To this purpose, GECs were seeded into 24-well plates (2 × 10^4^/well), grown until confluence, and then treated with the specific treatments for 72 h. Then, the mixture of Calcein AM and EthD-1 was prepared following manufacturer’s instruction, and administered to cell cultures. Fluorescence images were acquired with Eclipse Ti-E microscope (Nikon Instruments, Campi Bisenzio, Italy).

### 2.6. Annexin V Apoptosis

The assessment of apoptosis was performed using the RealTime-Glo™ Annexin V Apoptosis and Necrosis Assay, a live-cell (non-lytic) real-time (kinetic) assay that measures the exposure of phosphatidylserine (PS) on the outer leaflet of the cell membrane during the apoptotic process. GECs and GBM cells (5 × 10^3^/well) were seeded and cultured in 96-well plate for 24 h. Then, specific treatments were administered together with the detection reagent. Upon loss of membrane integrity, the dye entered the cell and bound to DNA, generating a fluorescent signal. Fluorescence was read with a microplate reader at 8 h, 24 h, 48 h and 72 h after treatments.

### 2.7. Caspase 3/7 Activity

Caspase 3/7 activity was evaluated by the luminescent assay Caspase-Glo^®^ 3/7 Assay. To this purpose, GECs and GBM cells (5 × 10^3^/well) were seeded and cultured in 96-well plate for 24 h and then submitted to specific treatments. After 72 h, Caspase-Glo^®^ 3/7 Reagent was added as an “add-mix-measure” format resulting in cell lysis, followed by caspase cleavage of the substrate and generation of a “glow-type” luminescent signal, proportional to the amount of caspase activity, produced by luciferase. Luminescence was read with a microplate reader.

### 2.8. Tube-like Structure Formation Assay

μ-Plate Angiogenesis 96-Well (Ibidi) were coated with 12.5 mg/mL Matrigel (BD Biosciences, Franklin Lakes, NJ, USA), 10 μL/well, at 4 °C. After gentle agitation to ensure complete coating, plates were incubated for 30 min at 37 °C to allow Matrigel solidification. GECs were then seeded (10^4^/well) and cultured in basal condition or with specific treatments. Cord formation was monitored with an inverted Eclipse Ti-E microscope (Nikon Instruments). After 24 h of incubation, five random images were acquired and analyzed with the “Angiogenesis Analyzer” plugin in ImageJ 26.

### 2.9. Migration Assay

GECs were seeded (1 × 10^4^ cells, each side) into Ibidi Culture-Inserts (Ibidi). and cultured until 95% confluence was reached. After that, the inserts were removed, and cells were stained with 1 mg/mL Calcein AM (Thermo Fisher Scientific) for 30 min at 37 °C. Then, fresh EndoPM was added in the presence of pharmacological treatments, as previously described. After 24 h, images of GECs that migrated into the cells-free gap were acquired with an inverted Leica DMI6000B widefield microscope at 20× magnifications in five random fields. Cells migrated into the gap were than counted using “Analyze Particles” in ImageJ.

### 2.10. Quantitative Real-Time PCR Analysis (qRT-PCR)

GECs (1 × 10^5^/well) were seeded into 25 cm^2^ collagen-coated culture flasks. When 90% confluence was reached, or at the end of the above listed treatment, total RNA was extracted following TRI-Reagent protocol and quantified with NanoDrop 1000 Spectrophotometer (Thermo Fisher Scientific). Reverse transcriptase reaction was executed using TranScriba Kit (A&A Biotechnology), loading 1 μg of RNA (A260/A280 > 1.8), according to the manufacturer’s instructions. qRT-PCR was performed using StepOnePlus™ (Thermo Fisher Scientific), 1 μg of cDNA, forward and reverse primers (250 nM each) (Table 2) and PowerUp SYBR Green Master Mix (Thermo Fisher Scientific). Data were normalized to 18S expression, used as endogenous control. Relative gene expression was determined using the 2^−ΔΔCt^ method.

### 2.11. Western Blot Analyses

GECs (2 × 10^5^) were seeded into a 25 cm^2^ collagen-coated culture flasks precoated with Collagen Bovine Type I and cultured until they reached the appropriate confluence (about 80–90%). Then, cells were lysed with M-PER Protein Extraction Reagent (Thermo Fisher Scientific) in the presence of Halt Protease Inhibitor Cocktail (Thermo Fisher Scientific). Proteins were quantified by the Pierce Detergent Compatible Bradford Assay Kit (Thermo Fisher Scientific). Protein lysates (30 mg) were separated in Bolt 10% Bis-Tris Plus Gels (Thermo Fisher Scientific) in Mini Gel Tank (Thermo Fisher Scientific) and transferred onto nitrocellulose iBlot 2 Transfer Stacks using iBlot 2 Dry Blotting System (Thermo Fisher Scientific). After transfer, the membrane was blocked in Tris-buffered saline/Tween 20 þ 5% milk solution and incubated separately with anti-RAS (Santa Cruz Biotechnology), anti-HIF1a (Thermo Fisher Scientific), anti-BAX (Thermo Fisher Scientific), anti-BCL2 (Thermo Fisher Scientific), anti-SPHK1 (Santa Cruz Biotechnology), anti-S1PR1 (Santa Cruz Biotechnology) and anti-CERS (Abcam), overnight at 4 °C. After incubation with HRP-labeled secondary antibody (Invitrogen, Carlsbad, CA, USA), protein bands were scanned with SuperSignal West Pico PLUS Chemiluminescent Substrate (Thermo Fisher Scientific) and detected by ChemiDoc XRSþ (Bio-Rad, Hercules, CA, USA). Densitometric analysis were performed using ImageJ.

### 2.12. Statistical Analyses

All analyses were done with GraphPad Prism (GraphPad Software, Inc., La Jolla, CA, USA). Parameters were compared and analyzed by a one-way analysis of variance. When significant differences were detected, Dunnet post hoc comparisons versus control group were made. Differences were considered statistically significant for *p* < 0.05.

## 3. Results

The clinical and molecular features of patients and their respective glioblastoma tissues are provided in Table 1. The median age of patients at diagnosis was 60 years (IQR: 47–76) and 67% were males. Five patients suffered from a tumor located in the temporal lobe, often resulting in cognitive dysfunctions such as personality changes, mood disorders, and short-term memory deficits [27]. The median value of MGMT promoter methylation was 14 (IQR: 6.5–31.5) and all glioblastomas were wildtype for IDH. Notably, a value of MGMT promoter methylation > 9% is considered a favorable prognostic indicator, associated with a better response to treatment [28].

**Figure 1:**
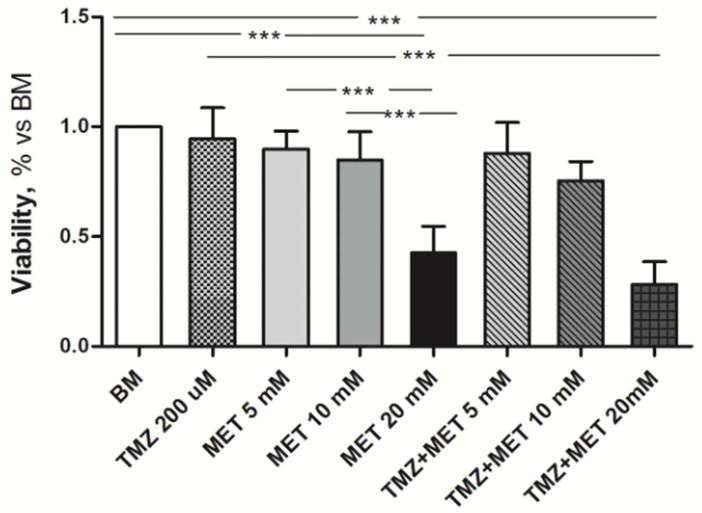
Effect of MET, alone or in combination with TMZ in scaling doses, on GEC viability, assessed by MTT after 72 h of treatment. Data are the mean ± SD of at least three experiments in triplicate. *** *p* < 0.001.

The assessment of GEC viability by MTT assay showed that the most significant effect of MET manifested at the concentration of 20 mM, with a 50% decrease in viable cells, whereas no appreciable effect was observed with MET at 5 mM and 10 mM. This result was confirmed by the combined treatment with TMZ, which exerted the highest efficacy with MET at 20 mM (Figure 1).

**Figure 2:**
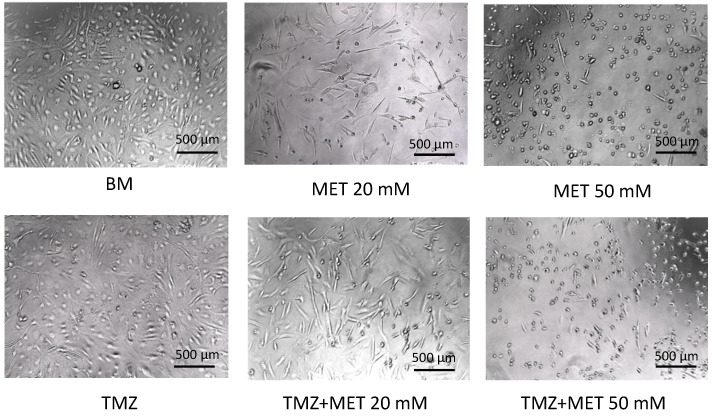
Representative images of GECs captured with optical microscope, after 48 h of treatment with MET, alone and in combination with TMZ. BM: Basal Medium, TMZ: Temozolomide, MET: Metformin. Scale bar: 500 µm.

To select the best concentration of MET in terms of cytotoxicity, we further increased the concentration to 50 mM, monitoring GEC viability by optical microscope. As seen in Figure 2, after 48 h of treatment, we observed a drastic increase in cell death after administration of MET 50 mM, as the detachment of GECs from the adhesion monolayer, the decrease in cell volume and several signs of necrosis.

**Figure 3:**
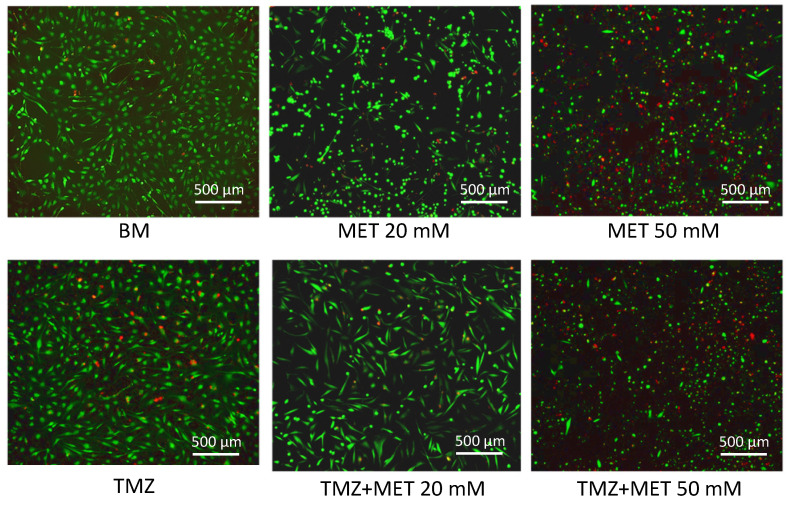
Representative images of GECs treated for 48 h with MET, alone and in combination with TMZ, captured after Live/Dead assay. The cells in green are alive, while the red ones are dead. BM: Basal Medium, TMZ: Temozolomide, MET: Metformin. Scale bar: 500 µm.

In order to confirm this observation, we performed a Live/Dead assay, to optimize the estimation of non-viable cells. The representative images in Figure 3 show viable cells in green fluorescence (Calcein AM) and dead cell in red (EthD-1), proving a severe increase of GEC death after treatment with MET 50 mM, administered both alone and in combination with TMZ.

**Figure 4:**
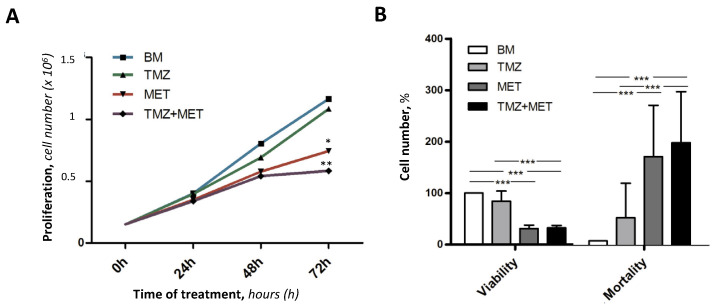
Effect of basal medium (BM), TMZ (200 μM), MET (20 mM), or combination of the two, on GEC proliferation (**A**) and viability (**B**) at different times (**A**), and at 72 h (**B**) of treatment. Number of viable and non-viable cell (as %) after 72 h of treatment. Data are the mean ± SD of at least 3 experiments in triplicate.* *p* < 0.05, ** *p* < 0.01, *** *p* < 0.001.

These preliminary results suggested to us to continue the experiments using the optimal concentration of MET 20 mM, to avoid necrotic events. Therefore, we evaluated MET effect on GEC proliferation. The results (Figure 4A) revealed that GECs in basal medium (BM) or treated with TMZ exhibited a linear growth trend, whereas GECs treated with MET, either alone or with TMZ, reached a plateau state between 48 h and 72 h. Further, at 72 h, we found a significant decrease in cell viability, and a parallel significant increase in mortality, in GECs treated with MET, alone and in co-administration with TMZ (Figure 4B).

**Figure 5:**
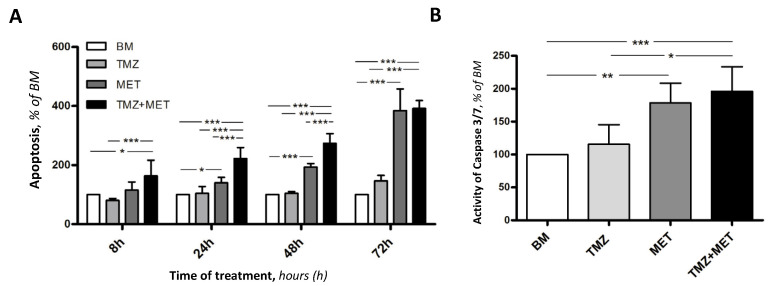
Effect of basal medium (BM), 200 μM temozolomide (TMZ), 20 mM MET, or the combination of the two (TMZ + MET), on GEC apoptosis. (**A**) Estimation of apoptosis by annexin V apoptosis assay at different times of treatment. (**B**) Caspase 3/7 activity at 72 h of treatment. Data are the mean ± SD of at least 3 experiments in triplicate * *p* < 0.05, ** *p* < 0.01, *** *p* < 0.001.

With the aim of combining the viability data with those relating to the cellular apoptosis, we evaluated the apoptotic mechanism by annexin V and caspase 3/7 activity (Figure 5). The annexin V assay allowed us to measure apoptotic events in real-time during the treatment protocol, thus we obtained data at 8 h, 24 h, 48 h, and 72 h. The efficacy of MET in inducing GEC apoptosis proved to be time-dependent, with statistically significant results of MET + TMZ versus BM and TMZ, already after 8 h from the beginning of the treatment. Over time, treatment with MET considerably increased the gap with both BM and TMZ, with the highest efficacy observed after 72 h of treatment with MET and MET + TMZ (Figure 5A). To confirm the activation of apoptotic process, we evaluated the activity of caspase 3/7. It has been demonstrated that cell death is more efficient in the presence of caspase-3, which is the primary executioner of apoptotic death, whereas caspase-7 causes an accumulation of reactive oxygen species (ROS) production and functions to detach cells from the extracellular matrix, thus playing a supportive role in the execution phase of apoptosis [29]. Our results showed a significant increase of the activity of complex caspase 3/7 in GECs treated with MET, alone or in combination with TMZ, supporting the efficacy of MET in inhibiting GEC survival (Figure 5B).

**Figure 6:**
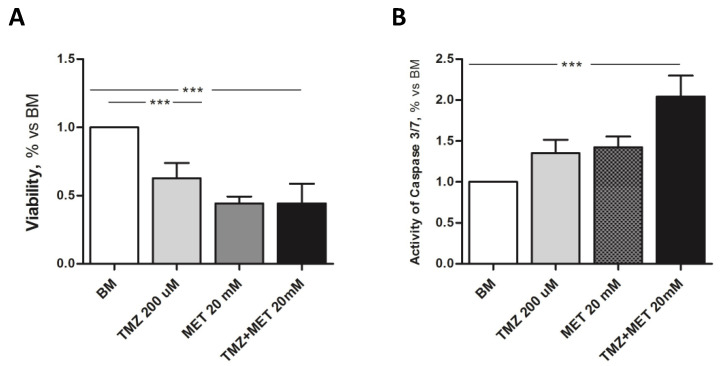
Effect of basal medium (BM), 200 μM temozolomide (TMZ), 20 mM MET, or the combination of the two (TMZ + MET), on GBM cells viability (**A**) and apoptosis (**B**), analyzed by the activation of caspase 3/7. Data are the mean ± SD of at least 3 experiments in triplicate, *** *p* < 0.001.

The effect of MET alone or in combination with TMZ was also tested on GBM cells, confirming the pro-apoptotic efficacy of MET, especially in combination with TMZ (Figure 6). Notably, GBM cells proved to be more sensitive to TMZ, corroborating the evidence that the heterogeneous cellular components of glioblastoma present a differential response to therapy, for which a multitargeted approach is needed.

**Figure 7:**
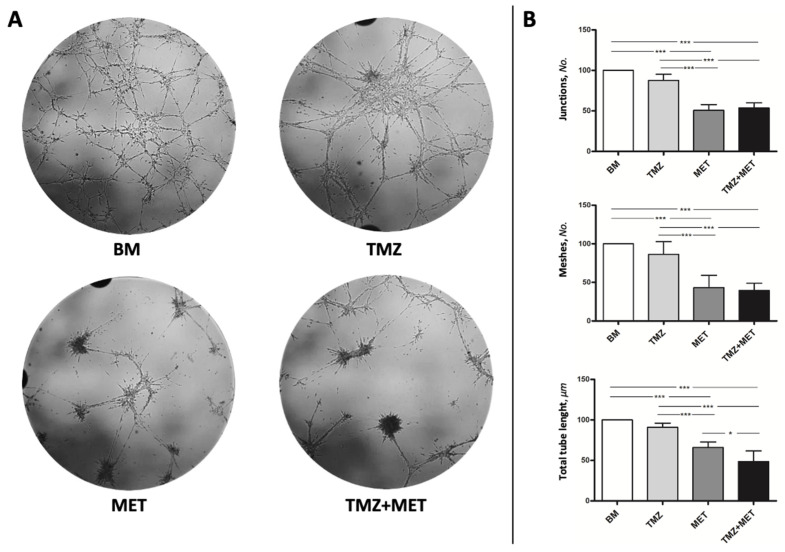
Effect of MET and TMZ on tube-formation by GECs. (**A**) Representative images of GECs cultured for 48 h in Matrigel with 200 μM TMZ, 20 mM MET or TMZ + MET. (**B**) Quantification of junctions, meshes, and total tube length measured using the Angiogenesis Analyzer plugin in ImageJ. Data are the mean ± SD of at least 3 experiments in triplicate. Magnification 10×. * *p* < 0.05, *** *p* < 0.001.

In order to evaluate the impact of MET on angiogenesis, we then performed a tube-like formation assay (Figure 7), which measures the cell ability to form capillary-like structures. When GECs were seeded on Matrigel, they gradually formed capillary-like tubular structures, connected to each other, creating a mesh-like configuration. We found that MET was able to significantly inhibit the formation of the tube network both alone and combined with TMZ treatment (Figure 7A). Quantitative analyses revealed that MET induced a significant decrease of the number of junctions, meshes, and tube length (Figure 7B).

**Figure 8:**
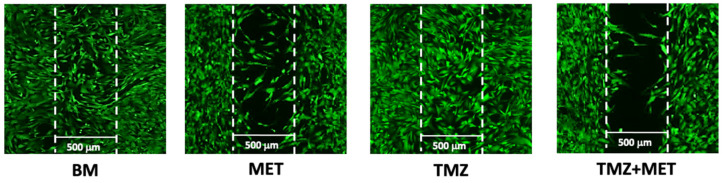
Effect of MET and TMZ on migration of GECs. Representative images of GECs cultured into Ibidi Culture-Inserts for 48 h with TMZ 200 μM, MET 20 mM and TMZ + MET.

In addition, migration assay revealed that the administration of MET, both alone and in combination with TMZ, resulted in a significant decrease of the number of cells migrated into the gap (Figure 8).

**Figure 9:**
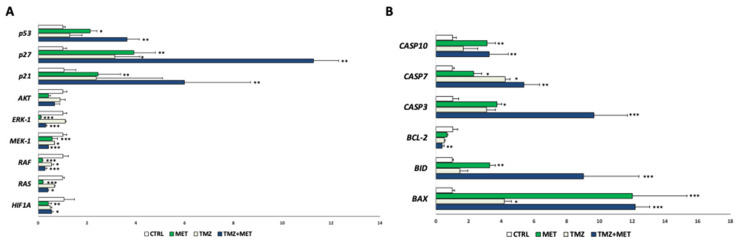
Gene expression in GECs after 48 h treatment with MET (20 mM), alone or in combination with TMZ by qRT-PCR of proliferative (**A**) and apoptotic mediators (**B**). Values are expressed as mean ± SD of at least 3 independent experiments. * *p* < 0.05 ** *p* < 0.01; *** *p* < 0.001.

Finally, with the aim of investigating a potential molecular mechanism underlying the effect of MET, a gene expression screening was performed. The results revealed a down-regulation of proliferative signaling pathways (MAPK, RAF/RAS/MERK/ERK, HIF-1a) and anti-apoptotic mediators (Bcl-2), as well an up-regulation of onco-suppressor genes (p53, p21 and p27) and pro-apoptotic mediators (Caspases and Bax-family) (Figure 9A,B).

**Figure 10:**
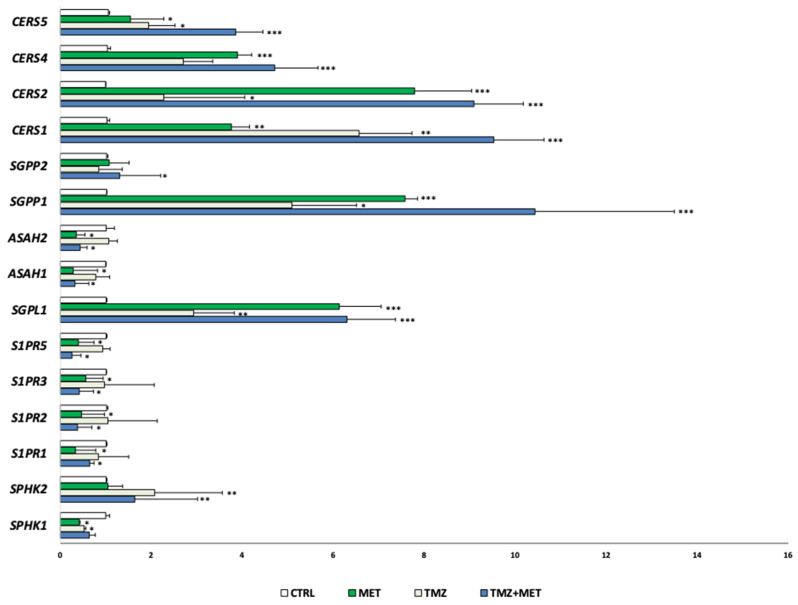
Gene expression in GECs after treatment with MET, alone or in combination with TMZ by qRT-PCR. * *p* < 0.05 ** *p* < 0.01; *** *p* < 0.001. CERS: ceramide synthase; SGPP: S1P phosphatase; ASAH: acid ceramidase; SGPL: S1P lyase; S1PR: S1P receptor; SPHK: sphingosine kinase.

In addition, and intriguingly, the evaluation of gene expression of sphingolipid metabolism and signaling revealed that MET treatment induced an up-regulated expression of genes encoding enzymes responsible for ceramide biosynthesis, and a down-regulation of mediators involved in S1P signaling (Figure 10).

**Figure 11:**
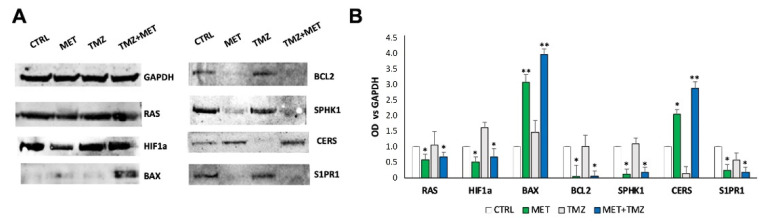
(**A**) Protein expression in GECs after treatment with MET, TMZ, and their combination by Western Blot. (**B**) Densitometric analysis in ImageJ was performed to quantify protein content, expressed by OD versus GAPDH, used to normalize the results for the total content of proteins. Data are the mean ± SD of 3 independent experiments with different primary GECs, run in triplicate. * *p* < 0.05, ** *p* < 0.01, versus untreated control (CTRL). The full western blot details are available in Figure S1.

Gene expression data were confirmed by protein analysis conducted by Western Blot on the key mediators of identified pathways, which support the downregulation of proliferative markers, the upregulation of pro-apoptotic mediators and the suppression of S1P oncopromoter signaling toward a pro-apoptotic one (Figure 11).

## 4. Discussion

Several studies performed in the last century demonstrated that MET was able to decrease fasting and post-fasting glucose, insulin resistance and glycated hemoglobin [14], leading to its use as a safe and high tolerable first line treatment of T2DM, hyperlipidemia, and non-alcoholic fatty liver disease [30,31]. In addition, and of relevance, recent epidemiological studies observed that MET administration to diabetic patients at the standard clinical dose, succeeded in reducing cancer incidence and/or related mortality, suggesting its potential anti-cancer effect, also against glioblastoma [15]. Preclinical studies on in vitro models confirmed the ability of MET in reducing tumor cell growth and proliferation in different cancers, both alone or in combination with chemo- and radio-therapy. Several observations have also been made in glioblastoma cell lines, where MET proved to synergize with TMZ, the standard therapeutic approach. Sesen et al. demonstrated that metformin decrease proliferation and induce cell cycle arrest, cell death, autophagy and apoptosis of human glioblastoma cells, with a decrease of mitochondrial-dependent ATP production and oxygen consumption and an increase lactate [18]. Similarly, Yu et al. proved that TMZ in combination with metformin act synergistically to inhibit proliferation and expansion of glioma stem-like cells, reducing Akt activation [19]. Several pre-clinical models reported the efficacy of metformin against glioblastoma cells, with different molecular mechanisms involving for example the chloride intracellular channel protein 1 (CLIC1) [32], the AMP-activated protein kinase (AMPK) [33], Akt and FOXO3 activation [34,35,36].

Furthermore, researchers of our teamwork previously found that MET enhances TMZ effect on TMZ-sensitive cell line (U251) and overcomes TMZ-resistance in T98G GBM cell line. This effect was mediated by the increase of pro-apoptotic mechanisms mediated by Bax family and the reduction of reactive oxygen species (ROS) production [37]. Further, the reduced viability of U251 cells treated with MET was found to be related with the reduction of hypoxia-inducible factor (HIF-1α) and vascular endothelial growth factor (VEGF), key actors of glioblastoma angiogenesis, as well as with the inhibition of PI3K/mTOR axis [20].

However, the main limit of previous studies may be the use of commercial cell lines, which did not account for the molecular characteristic of individual patients. Here, we tested the effect of MET in patient-specific ex vivo models, suggesting the potential of early prediction of patient response to a combined treatment, thus optimizing the therapeutic approach. Of relevance, we used a peculiar model, the endothelial glioblastoma component, whose contribution in glioblastoma progression and therapy resistance is now clearly established, and under investigation for anti-angiogenic target therapy.

Our data demonstrate that MET is able to inhibit GEC proliferation with a time- and dose-dependent trend, suggesting the possibility to adjust the therapeutic administration to optimize safety, tolerability, and patient’s outcome. Furthermore, the proved ability of the optimal dose of MET in promoting cell apoptosis by activating caspases, without producing necrotic events, may imply the specificity against GECs presenting aberrant proliferation. In GBM cells, the ability of MET to decrease cell viability and induce apoptosis was confirmed, but in this case, we observed a higher efficacy of TMZ, which was exacerbated by the combined treatment. These data confirmed the heterogeneity of GBM cell components, whose different response to therapy corroborates the necessity of a multitargeted therapeutic approach.

The gene expression screening confirmed the inhibition of proliferative pathways, as those mediated by Akt and MAPK Raf, Ras, Mek-1, and Erk-1, as well the decrease of anti-apoptotic mediators as Bcl-2. These results were concomitant to the up-regulation of caspases, pro-apoptotic mediators, as Bax and Bid, and tumor suppressor genes as p53, p21 an p27.

The Ras/RAF/MEK/ERK (MAPK) signaling represent one of the best-characterized pathways in cancer biology, and its hyperactivation is associated to more than 40% of human cancers. The signaling by MAPK promotes cellular overgrowth by turning on proliferative genes, and simultaneously enables cells to overcome metabolic stress by inhibiting AMPK signaling. Mechanistically, upon binding of RTKs or other stimulations, Ras small GTPases are activated by GTP/GDP exchange factors (GEFs), which in turn recruit RAF/MEK complexes to the plasma membrane, and trigger the RAF/MEK/ERK kinase cascade through facilitating RAF/RAF (or KSR), RAF/MEK, and MEK/MEK interactions as well as subsequent phosphorylation [38]. Active ERKs are further translocated into the nuclei or in the cytoplasm, where they phosphorylate a number of substrates that regulate cell proliferation and survival [39,40,41]. An aberrant activation of MAPK signaling frequently induces human cancers, including glioblastoma [42,43]. Our data revealed that the downregulation of MAPK was accompanied by the decreased expression of Bcl-2, the family of proteins regulating all major types of cell death (including necrosis, autophagy and apoptosis), and thus operating as nodal points at the convergence of multiple pathways with broad relevance to oncology [44]. This result is further confirmed by the overexpression of Bax and Bid, pro-apoptotic members of the Bcl-2 family, and of caspase-3 and -7, the major executioner caspases of apoptosis [45]. Interesting data also arise from the MET-induced upregulation of p53, p21 and p27. The *TP53* encodes a transcription factor that is a critical barrier to carcinogenesis. Inactivation of TP53 is the most common mutation in sporadic human cancers, suggesting a strong selection against p53 function during tumorigenesis. p53 is thought to act as a tumor suppressor by serving as a cellular stress sensor. The inheritance of a mutant *TP53* allele is observed in Li-Fraumeni syndrome, predisposing patients to early onset of cancer development, further underscoring the role of p53 in tumor suppression [46]. Similarly, p21 functions as a cell cycle inhibitor, an anti-proliferative effector in normal cells and is dysregulated in some cancers. P27 is considered a tumor suppressor because of its function as a regulator of the cell cycle. In cancers it is often inactivated via impaired synthesis, accelerated degradation, or mislocalization [47].

Notably, these reported effects of MET were enhanced by the co-administration with TMZ, advancing the promising synergistic anti-cancer activity of MET as add-on therapy.

**Figure 12:**
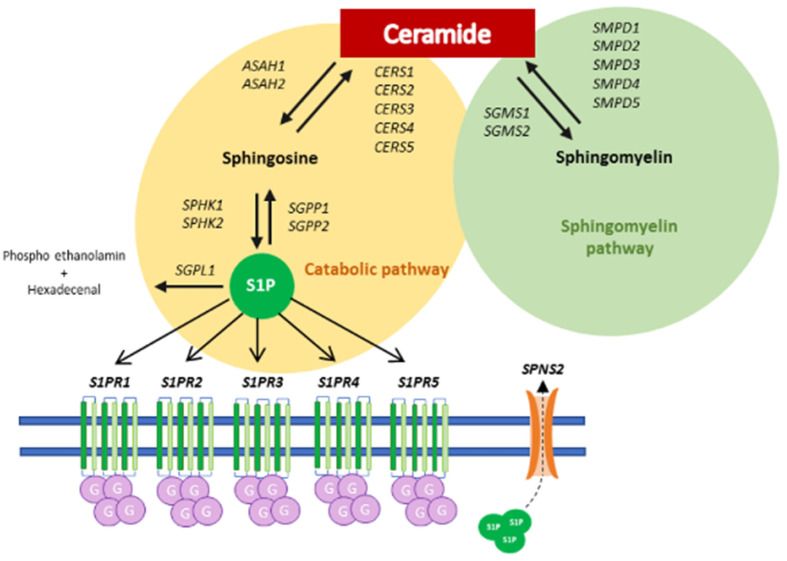
Schematic representation of S1P and Cer metabolism and signaling, with the relative enzymes involved. SphK: sphingosine kinase; S1PRs: S1P receptors; SGPL: S1P lyase; SGPP: S1P phosphatase; ASAH: acid ceramidase; CERS: ceramide synthase; SGMS: sphingomyelin synthase; SMPD: sphingomyelin phosphodiesterase; SPNS2: sphingolipid transporter 2.

The exploration of scientific literature reporting MET activity disclosed its effect in influencing sphingolipid metabolism, improving oxidative stress status [21], as well as the role of sphingolipids, especially sphingosine-1-phosphate (S1P), in T2DM [48], prompting the idea of a potential unrevealed target of MET in cancer. Our previous observation demonstrated the high contribution of sphingolipids in cancer progression, as S1P represents an active oncopromoter lipid with pleiotropic functions [23,24,49]. In particular, S1P is able to stimulate cellular processes strictly related to cancer, as proliferation, invasion, survival and angiogenesis [50]. The biosynthesis of S1P occurs during complex sphingolipid catabolism, via the conversion of ceramide (Cer) to sphingosine, which is then phosphorylated to S1P by sphingosine kinases (SphK1/2) [51]. S1P exerts its effects as extracellular signal by binding five specific G protein-coupled receptors (S1PR1–5), located in plasma membrane [51] (Figure 12). Cer and S1P are therefore two interconvertible lipids, and are able to control cell fate in an opposite fashion. In particular, Cer favors anti-proliferative and cell death pathways such as senescence and apoptosis, whereas S1P stimulates cell proliferation and survival pathways. The balance between these opposing signaling functions is referred to as the “sphingolipid rheostat” and is slightly modulated by the expression and activity of intermediary enzymes. A shift in this balance toward S1P results in tumor cell survival and resistance to chemotherapy, whereas a shift toward Cer production results in cell apoptosis.

Our previous studies reported that S1P plays pleiotropic functions in gliomagenesis [23,26,49,50]. In particular, we demonstrated that fast-proliferating glioblastoma stem cells (GSCs) perform a rapid degradation of newly synthesized Cer and exhibit a faster flux converting Sph to S1P, with a 10-fold higher release of S1P in the extracellular milieu [23], compared to slow-proliferating GSCs. Furthermore, we found that, co-cultured with glioblastoma cells, GECs exhibit increased SphK2 expression and activity, with a significant S1P secretion enhancement. In turn, in an autocrine/paracrine manner, the extracellular S1P stimulates glioblastoma cell growth and GEC migration and tubule formation in a S1PR1/S1PR3-dependent trend [26]. In addition, platelets represent the main source of circulating S1P, due to the high SphK activity [52] and lack S1P lyase [53], so that their recruitment and activation on GEC surface lead to the release of S1P in glioblastoma microenvironment, which in turn exacerbate glioblastoma aggressiveness.

In this contest, we found that MET determines the up-regulation of genes coding enzymes that mediate the catabolic process of S1P into sphingosine, the S1P phosphatases, as well as those responsible for Cer production, as the Cer synthases. This effect was accompanied by the simultaneous down-regulation of the enzyme mediating Cer degradation, as the acid ceramidases, and S1P production and signaling, i.e., S1P kinases, and S1P receptors. These data suggest a sphingolipid-related activity of MET, whose anti-proliferative and pro-apoptotic effects may be due to the shift in the sphingolipid rheostat in favor of Cer. Our data are in line with the observations by Hart et al., which demonstrated that MET blocks hypoxia-induced SphK1, whose high expression was found to promote ovarian cancer progression [54].

Overall, our data support MET efficiency as add-on therapy for the treatment of glioblastoma patients, and the rationale for a combined therapeutic approach involving a totally safe and tolerable drug, whose optimal dose needs to be adjusted to obtain the best clinical outcome [55].

However, it should be mentioned that the results of some clinical trials consisting in the administration of MET in glioma patients deserve attention for their controversial outcomes. A recent clinical trial assessed MET efficacy as neo-adjuvant compound together with TMZ and hypofractionated accelerated radio-therapy (HART) in 33 patients with glioblastoma. The study confirmed no adverse effects after the use of MET, confirming its safety and tolerability and validating previous results on favorable outcomes of glioblastoma patients, particularly those with low methylation levels of MGMT (NCT02780024) [56]. However, Seliger et al. observed that MET does not succeed in increasing glioblastoma patient survival, neither alone nor in combination with other drugs [57]. With the purpose to better comprehend the anti-cancer potential of MET, a phase II clinical trial by the Weill Medical College of Cornell University is actually recruiting glioblastoma patients to evaluate the tolerability and the effects of a ketogenic diet in conjunction with MET (NCT04691960). In addition, a recent multicentric phase II clinical trial conducted by the Hospital Foch and the National Cancer Institute in France and named OPTIMUM, is recruiting 640 participants with *IDH*-wildtype glioblastoma, starting from the observed overexpression of mitochondrial markers in *IDH-wt* glioblastomas undergoing oxidative stress (NCT04945148).

## 5. Conclusions

Taken together, the results of this study advance novel insights in the potential use of MET in oncology, suggesting a promising translational relevance, which needs further confirmation and correlation with molecular parameters. The identification of predictive biomarkers of therapy response, and the identification responsive glioblastoma subtypes may really impact clinical practice in terms of personalized therapeutic approaches.

## Figures and Tables

**Table 1:** Clinical and molecular data of glioblastoma patients.

Sample ID	Age	Sex	Tumor Location	KPS	IDH	MGMT	MIB-1
Poli 09	81	F	FP left	60	wt	4%	15%
Poli 182	41	F	T left	70	wt	49%	55%
Poli 183	41	M	T right	80	wt	14%	40%
Poli 187	45	M	F right	100	wt	37%	20%
Poli 208	60	M	P right	80	wt	14%	30%
Poli 210	52	M	P left	70	wt	15%	55%
Poli 214	66	M	T left	80	wt	60%	65%
Poli 230	82	F	T left	70	wt	3%	27%
Poli 231	80	M	T right	80	wt	2%	40%

KPS: Karnofsky Performance Status; IDH: Isocitrate dehydrogenase; MGMT: O6-methylguanine (O6-MeG)-DNA methyltransferase; MIB-1: Mindbomb Homolog-1 Index; FP: fronto-parietal lobe; T: temporal lobe; F: frontal lobe; P: parietal lobe; wt: wild-type.

**Table 2:** List of primer sequences (5′-3′ and 3′-5′) with relative melting temperature.

Gene	Forward Primer (5′-3′)	Reverse Primer (3′-5′)	Tm (°C)
*18 S*	ACTTTCGATGGTAGTCGCCGT	CCTTGGATGTGGTAGCCGTTT	61
*AKT*	TCT ATG GCG CTG AGA TTG TG	CTT AAT GTG CCC GTC CTT GT	58
*BAX*	AGC AAA CTG GTG CTC AAG G	TCT TGG ATC CAG CCC AAC	57
*BCL-2*	AGT ACC TGA ACC GGC ACC T	GCC GTA CAG TTC CAC AAA GG	60
*BID*	ACC GTG GTC TTT CCA GCA CC	TCT GCG GAA GCT GTT GTC AG	61
*SPHK1*	TGCAGTTGGTCAGGAGGTCT	GCTCTGGTGGTCATGTCTGG	66
*SPHK2*	CCCCGGTTGCTTCTATTGGT	ATCCCACTCACTCAGGCTCA	66
*S1PR1*	GGGAGCAATAACTTCCGCCT	AAGCAGAGTGAAGACCGTGG	66
*S1PR2*	CCTGTACGTGCGCATCTACT	GCTTTGTAGAGGATCGGGCA	65
*S1PR3*	CAACCACAACAACTCGGAGC	GCCAACACGATGAACCACTG	64
*S1PR5*	CATCTACTGCCAGGTACGCG	GAGCAACAGCAGCAGGAAGA	65
*SGPL1*	AAGCATATCGGGATCTGGCC	TAGCTCTTCTCATTGCCCGC	65
*SGPP1*	CGTGGTCAAGTTGGAGGTCT	GGCAAACTAGAGAACACCAGC	63
*SGPP2*	AGGATGTCTTGAAGTGGCCC	CCATCACCAGTCCCAACACA	66
*ASAH1*	TTC TTT GCC TCT GCT GGA GTC	TGG AAC TGC ACC TCT GTA CG	60
*ASAH2*	CAT GGC AGA ACC TGA TGG GT	GTC TGT TCA GGA CCT CCA GC	61
*CERS1*	CCC TTC TTC CAT GAC CCA CC	CTC AGT GGC TTC TCG GCT TT	61
*CERS2*	TTT GCC CCT CAC TCA GGA TG	CGT AGA CAC GTC CAT CTC GG	61
*CERS4*	AGG AGC AGA GTC CGG CTG	CCT GCC AAA ACC ACT CGT TG	60
*CERS5*	GCT CTT CGA GCG ATT TAT TGC C	ATT CAC CCG ATT GGC ACC AT	60
*CASPASE-3*	ATG GTT TGA GCC TGA GCA GA	GGC AGC ATC ATC CAC ACA TAC	60
*CASPASE-7*	GAG CAG GGG GTT GAG GAT TC	GTC TTT TCC GTG CTC CTC CA	61
*CASPASE-10*	CCA GGT GAA CTG GAA TGC CT	CCA CTA GCT TTT GGC CCT GA	60
*ERK-1*	ACTCCAAAGCCCTTGACCTG	CTTCAGCCGCTCCTTAGGTA	60
*HIF-1a*	GTCTGAGGGGACAGGAGGAT	GCACCAAGCAGGTCATAGGT	61
*MEK-1*	CTTCGCAGAGCGGCTAGG	AGCTCTAGCTCCTCCAGCTT	61
*P21*	AGT ACC CTC TCA GCT CCA GG	TGT CTG ACT CCT TGT TCC GC	61
*P27*	TGG CTT GTC AGG AAC TCG AC	CTA GTC TCC AGG GAG GTG CT	63
*P53*	AGG CCT TGG AAC TCA AGG AT	CCC TTT TTG GAC TTC AGG TG	58
*RAF*	GGT TTT GGC GTA GAT TCC CC	ACC TGA AGC AAA GAT GGC GT	59
*RAS*	AGCAGGTGGTCATTGATGGG	CCGTTTGATCTGCTCCCTGT	60

## Data Availability

The data presented in this study are available in this article.

## Supplementary Materials

The following supporting information can be downloaded at: https://www.mdpi.com/article/10.3390/cancers14061412/s1, Figure S1: Full Western blot for Figure 11.
