# Supplementary material for: Effects of Metformin as Add-On Therapy against Glioblastoma: An Old Medicine for Novel Oncology Therapeutics
Source: Cancers (Basel). 2022 Mar 10;14(6):1412. doi: 10.3390/cancers14061412 (PMC8946812; doi:10.3390/cancers14061412)
Supplement: Supplementary file 1 [file cancers-14-01412-s001.zip › cancers-1626669-supplementary.pdf]

Supplementary Materials

Effects of Metformin as Add-On Therapy Against Glioblastoma: An Old Medicine for Novel Oncology Therapeutics

Laura Guarnaccia, Stefania E. Navone, Matteo M. Masseroli, Melissa Balsamo, Manuela Caroli, Silvia Valtorta, Rosa M. Moresco, Rolando Campanella, Luigi Schisano, Giorgio Fiore, Valentina Galiano, Emanuele Garzia, Giuseppe C. Appiani, Marco Locatelli, Laura Riboni and Giovanni Marfia

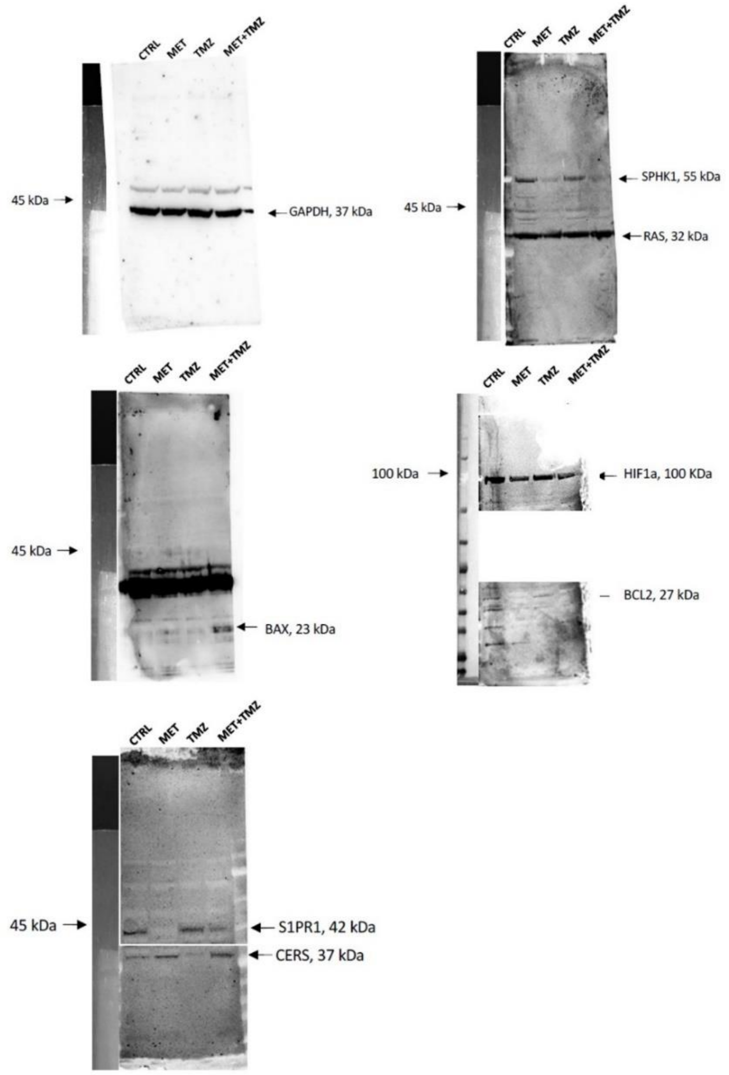

Figure S1. Full Western blot for Figure 11.

© 2022 by the authors. Licensee MDPI, Basel, Switzerland. This article is an open access article distributed under the terms and conditions of the Creative Commons Attribution (CC BY) license (http://creativecommons.org/licenses/by/4.0/).
